# Cartilage evaluation by ultrasonography in patients with rheumatoid arthritis: a scoping review

**DOI:** 10.1186/s41232-023-00286-2

**Published:** 2023-07-04

**Authors:** Takehisa Ogura, Takaharu Katagiri, Hideto Kameda

**Affiliations:** Department of Internal Medicine, Division of Rheumatology, Faculty of Medicine, Toho University, 2-22-36, Ohashi, Meguro-Ku, Tokyo, 153-8515 Japan

**Keywords:** Cartilage, Ultrasonography, Rheumatoid arthritis, Scoping review

## Abstract

**Background:**

This study aimed to provide an overview of ultrasonographic cartilage evaluation in patients with rheumatoid arthritis (RA) and identify research gaps in the utilization of cartilage evaluation.

**Methods:**

The study was conducted in accordance with the Preferred Reporting Items for Systematic Reviews and Meta-Analyses Extension for Scoping Reviews guidelines. A systematic literature search of the PubMed, Embase, and Cochrane Library databases was conducted for articles published up to July 2022 using the search term variations of “cartilage,” “ultrasonography,” and “rheumatoid arthritis.” Studies that included patients with RA who underwent cartilage evaluation by ultrasonography were selected. Articles published in languages other than English and about juvenile idiopathic arthritis were excluded.

**Results:**

Twenty-nine articles were identified. Most were cross-sectional studies (86%), mainly involving the metacarpophalangeal (55%) and knee (34%) joints. Assessments were performed using quantitative, binary, and semi-quantitative methods in 15, 10, and 15 studies, respectively. Reliability assessments were conducted in 10 studies, which showed feasible reliability but were limited to the finger joints. The validity assessment was validated in one study each that compared cartilage thickness measurements with cadaveric specimens and histological and semi-quantitative methods with surgical specimens, respectively. Comparisons with conventional radiography were also performed in six studies, which showed significant correlations. However, there was heterogeneity in the examination and assessment methods, and no adequate longitudinal evaluation was conducted.

**Conclusion:**

This review highlights the need for further research and validation of ultrasonographic cartilage assessment in patients with RA.

**Supplementary Information:**

The online version contains supplementary material available at 10.1186/s41232-023-00286-2.

## Background

Rheumatoid arthritis (RA) is a systemic autoimmune disease that predominantly involves the peripheral joints. It is characterized by the inflammatory proliferation of the synovium of the joints. Persistent synovitis causes bone and cartilage damage, leading to joint destruction and deformity.

To date, joint destruction has mainly been evaluated using conventional radiography (CR), which is simple, inexpensive, and widely used worldwide [1]. With CR, bone destruction can be evaluated through bone erosion and cartilage destruction based on joint space narrowing (JSN); however, early onset and minute changes are difficult to detect. Therefore, early diagnosis and monitoring of therapeutic targets using modern therapeutic strategies, such as biological disease-modifying antirheumatic drugs, may not be sufficient. In contrast, high-sensitivity imaging examinations such as magnetic resonance imaging (MRI) and ultrasonography (US) have been shown to detect joint damage earlier and with higher sensitivity [2]. Cartilage damage can particularly be evaluated indirectly by CR but can be evaluated directly using MRI and US [3, 4].

Compared with MRI, US is easier, less expensive, and has a higher resolution; therefore, it is considered useful for cartilage evaluation, including small joints. To date, cartilage evaluation using US has mainly been performed by quantitative evaluation based on thickness measurement and by binary evaluation based on the presence or absence of cartilage damage or graded semi-quantitative evaluation. However, in previous studies [5], the evaluation methods varied, and it is unclear which method is valid and valuable. Data are also lacking on their distinguishing abilities from other cartilage-damaging diseases, such as osteoarthritis (OA) and their usefulness as a monitoring tool in patients with RA. Therefore, the usefulness of cartilage evaluation by US in RA has not been fully clarified, which is one of the reasons why it is not used in daily clinical practice. This scoping review aimed to provide a current overview of cartilage evaluation by US in RA and identify research gaps in the utilization of cartilage evaluation.

## Methods

The study methodology was conducted in line with the Preferred Reporting Items for Systematic Reviews and Meta-Analyses Extension for Scoping Reviews guidelines [6, 7]. The preregistered protocol was not submitted prior to this review.

### Search strategy

The following bibliographic databases were screened: PubMed (from inception to July 2022), Embase (from inception to July 2022), and Cochrane Library (from inception to July 2022). The search terms included were variations of “cartilage,” “ultrasonography,” and “rheumatoid arthritis.” The final search formulae are presented in the supplementary data. All citations were imported into the web‐based bibliographic manager, RefWorks 2.0 (RefWorks‐COS, Bethesda, MD, USA), and duplicate citations were removed manually for the subsequent title and abstract relevance screening and data characterization of full articles.

### Study selection and data extraction

First, two authors independently reviewed the titles and abstracts of the identified studies. Second, the full text of each study that was deemed relevant was retrieved and independently reviewed by the two authors. Each author compiled a list of studies that met the inclusion and exclusion criteria. The lists were compared, and disagreements were resolved through discussion and consensus. Peer-reviewed articles that included patients with RA whose hyaline cartilage was examined ex vivo using US were selected. Studies involving juvenile idiopathic arthritis were excluded because the cartilage of adults and children are largely different [8, 9]. Case reports, review articles, letters to the editor, and conference abstracts were excluded from the analysis. In addition, articles published in languages other than English were excluded because of the limited resources for translation.

The data were extracted by one researcher and subsequently validated by a second researcher. Disagreements were resolved through discussions. For each selected study, the following data were extracted: study design, patient characteristics, joints assessed, evaluation methods, reliability, validity, and US techniques.

## Results

**Fig. 1:**
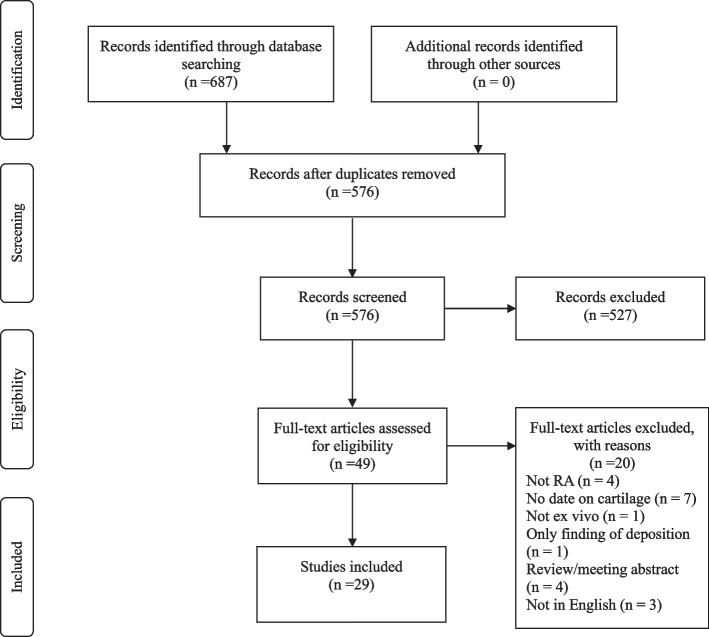
Flow diagram of the article selection

Figure 1 shows the flow diagram of the article selection process. A total of 687 citation records were screened from PubMed (*n* = 207), Embase (*n* = 468), and the Cochrane Library (*n* = 12). No citations were obtained by cross-referencing or related article searches. After removing duplicates, articles were screened based on the inclusion and exclusion criteria by reviewing the titles and abstracts. Forty-nine full-text articles were reviewed, of which 29 were included in the final analysis.

### Study characteristics

Table 1 shows the characteristics of the 29 included studies. One interventional study (3%) assessed changes over 52 weeks [10], whereas others were observational studies. Most of these were cross-sectional studies (25/29, 86%); one was a longitudinal study (assessed changes over 12 months) [11], and two (7%) [12, 13] were descriptive studies that did not compare or contrast. Six studies (21%) [3, 10, 14–17] were multicenter studies with more than one study participant or US assessor, and the remaining 23 (79%) were single-center studies. Fourteen studies (48%) performed non-cartilage assessments, such as synovial proliferation, intra-articular blood flow assessment, and bone erosion [10, 11, 14, 15, 18–27].

**Table 1:** Study characteristics

Author	Year	Country	Study designs	Single or multicenter study	Participants	No. of parents	Age, yr (median)	Sex (%female)	Disease duration, yr (median)	Joints
Aisen et al. [12]	1984	USA	D	S	RA + PsA/OA/others/healthy	2 + 1/7/3/7	48/67/25/31	—	—	Knee
Iagnocco et al. [28]	1992	Italy	CS	S	RA/OA/healthy	48/60/30	24–45/52–75/18–63 (range)	63/55/53	—	Knee
Grassi et al. [18]	1993	Italy	CS	S	RA/healthy	20/20	57/—	70/—	18.5/—	MCP 2 and 3
Østergaard et al. [19]	1995	Denmark	CS	S	RA/others/OA/healthy	10/3/2/5	49 (RA + others)/53/29	—	4 (RA + others)/8/—	Knee
Lund et al. [20]	1995	USA	CS	S	RA/healthy	29/0	24–74/28–56 (range)	90/50	0.5–2/— (range)	MCP 2–5, wrist
Batalov et al. [13]	2000	Bulgaria	D	S	RA/OA/healthy	30/14/14	36–57/36–73/25–37 (range)	—	—	Knee
Möller et al. [29]	2009	Switzerland	CS	S	RA/OA/others/healthy	48/18/24/34	RA (early: 45, persistent: 57)/59/50/42 (median)	75/89/63/68	RA (early: 0.3, persistent: 8)/1/0/— (median)	MCP 2–5, PIP 2–5
Darweesh et al. [21]	2010	India	CS	S	RA/OA	32/10	42/59	75/67	7.8/10.5	Knee
Sanja et al. [22]	2010	Serbia	CS	S	RA/DSD	77/101	61/61	70/63	7.3/—	Shoulder
Filippucci et al. [30]	2010	Italy	CS	S	RA	20	51	80	8.7	MCP 2 and 3
Riente et al. [14]	2010	Italy	CS	M	RA	100	58	79	8.0	Knee
Yücesoy et al. [23]	2011	Turkey	CS	S	RA	30	56	83	—	Knee
Di Geso et al. [15]	2012	Italy	CS	M	RA	52	62	75	9.7	Hip
Pereira et al. [24]	2014	Brazil	CS	S	RA	60	58	—	16.4	MCP 1–5
Bisi et al. [25]	2014	Brazil	CS	S	RA	60	58	78	10.0	MCP 2 and 3
Mandl et al. [3]	2015	Austria	CS	M	RA/healthy (cadaver)	35/5	63/78	83/100	10.6/—	MCP 2–5, PIP 2–5
Onodera et al. [31]	2015	Japan	CS	S	RA	15	66	87	—	MTP 2–5
Luz et al. [11]	2016	Brazil	L	S	RA	48	48	100	0.6	MCP 2 and 3
Mesci et al. [32]	2016	Turkey	CS	S	RA/healthy	40/40	54/51	88/85	6/— (median)	Knee
Sakthiswary et al. [33]	2017	Malaysia	CS	S	RA/healthy	61/27	53/49	90/89	8.8/—	Knee
Sarzi-Puttini et al. [10]	2018	Italy	I	M	RA	66	53	82	1.5 (median)	MCP 2 and 3, most clinically involved MCP
Hurnakova et al. [16]	2019	Italy	CS	M	RA/OA	52/34	58/64	71/79	9/4.7	MCP 2–5
Yang et al. [26]	2019	China	CS	S	RA/healthy	53/30	42/42	83/77	0.9/—	MCP 1–5, PIP 1–5, wrist
Mandl et al. [17]	2019	Austria	CS	M	RA	6	64	67	15	MCP 2–5, PIP 2–5
Abda et al. [27]	2020	Egypt	CS	S	RA	100	45	88	—	MCP 2 and 3
Ogura et al. [34]	2021	Italy	CS	S	RA/healthy	103/42	65/63 (median)	77/86	5.8/— (median)	MCP 2–5, PIP 2–5
Cipolletta et al. [35]	2020	Japan	CS	S	RA/healthy	20/15	50/56	75/67	10.7/—	MCP 2–5
Cipolletta et al. [36]	2022	Italy	CS	S	RA/healthy	51/40	51/49	73/73	5.8/—	MCP 2–5
Yildirim et al. [37]	2022	Turkey	CS	S	RA/healthy	55/55	47/45	75/75	0.5/—	Knee, ankle

—, data not available; *CS*, cross-sectional study; *D*, descriptive study; *DSD*, degenerative shoulder disease; *I*, interventional study; *L*, longitudinal study; *M*, multicenter study; *MCP*, metacarpophalangeal; *MTP*, metatarsophalangeal; *OA*, osteoarthritis; *PIP*, proximal interphalangeal; *PsA*, psoriatic arthritis; *RA*, rheumatoid arthritis; *S*, single-center study

The metacarpophalangeal (MCP) joint was the most commonly assessed joint, with 16 (55%) studies assessing it [3, 10, 11, 16–18, 20, 24–27, 29, 30, 34–36]. Five studies assessed the proximal interphalangeal (PIP) joint [3, 17, 26, 29, 34]. Two studies also assessed the wrist but only described observations from the dorsal aspect; moreover, it was unclear which wrist cartilage was assessed [20, 26]. Only one study has evaluated the metatarsophalangeal (MTP) joint [31]. Regarding large joints, ten studies (34%) assessed the knee joint [12–14, 19, 21, 23, 28, 32, 33, 37]. Other studies included one each for the shoulder [22], foot [37], and hip joints [15], respectively.

There were 1974 study participants, of whom 1323 had RA, 374 were healthy (including five cadavers), 145 had OA, and 132 had other diseases. The median (range) number of participants in each study was 60 (6–178), while the number of patients with RA was 48 (2–103). The participating patients with RA had a mean or median age of 40–60 years. The mean or median duration of disease was less than 1 year to a maximum of 18.5 years. There was also a high proportion of women in all studies.

### US techniques

Table 2 lists the US scanning techniques used in the studies. One study used an undetailed probe [12], another used a sector probe [18], and all the others used linear probes. In studies involving fingers, one study in the 1990s used 5 or 7.5 MHz [20], whereas others used 13–22 MHz as the probe frequency. Studies involving large joints, such as the knee, shoulder, and hip joints, used probes at 5–14 MHz (one study was not mentioned [33]).

**Table 2:** Ultrasonography techniques

Author	US probe frequency (MHz)	US probe	Examination time	Joint position	Probe position (place/direction)	Orthogonal insonation angle
Aisen et al. [12]	7.5	—	—	Maximal flexion	Suprapatellar*/LS, TS	—
Iagnocco et al. [28]	5	Linear	—	Maximal flexion (about 120°)	Suprapatellar*/LS, TS	—
Grassi et al. [18]	13	Sector	—	Flexed ≧ 45°	Dosal†/L	—
Østergaard et al. [19]	7.5	Linear	—	Maximal flexion	Suprapatellar*/TS	Yes
Lund et al. [20]	5/7.5	Linear	—	Flexed 10–20°	Dosal†/LS	—
Batalov et al. [13]	7.5	Linear	—	Maximal flexion	Suprapatellar*/LS, TS	—
Möller et al. [29]	10–15/6–18	Linear	Within 5 min	Maximal flexion (90°)	Dosal†/LS	—
Darweesh et al. [21]	13	Linear	—	Maximal flexion	Suprapatellar*/TS	—
Sanja et al. [22]	7.5	Linear	—	—	—‡	—
Filippucci et al. [30]	6–18	Linear	Less than 5 min	Maximal flexion > 45°	Dorsal, volar†/LS, TS	—
Riente et al. [14]	10–14	Linear	—	Maximal flexion	—*	—
Yücesoy et al. [23]	7–12/6–11	Linear	—	Maximal flexion	—*/TS	—
Di Geso et al. [15]	6–8	Linear	—	Neutral position	anterior§/LS	—
Pereira et al. [24]	6–18	Linear	—	—	Dosal†/TS	—
Bisi et al. [25]	18	Linear	—	—	Dosal†/LS, TS	—
Mandl et al. [3]	9–15	Linear	—	Maximal flexion (90°)	Dosal†/LS	Yes
Onodera et al. [31]	5–13	Linear	—	—	plantar||/LS	—
Luz et al. [11]	6–18	Linear	—	—	Dosal†/—	—
Mesci et al. [32]	5–10	Linear	—	Maximal flexion	Suprapatellar*/TS	—
Sakthiswary et al. [33]	NA	Linear	—	Maximal flexion	Suprapatellar*/TS	—
Sarzi-Puttini et al. [10]	6–18	Linear	—	—	—†	—
Hurnakova et al. [16]	22	Linear	—	Maximal flexion	Dosal/L, TS	—
Yang et al. [26]	6–15	Linear	—	—	—†	—
Mandl et al. [17]	8–18/10–22	Linear	—	Maximal flexion	Dosal†/LS, TS	Yes
Abda et al. [27]	10–19	Linear	—	—	Dosal†/—	—
Ogura et al. [34]	7–14	Linear	—	Maximal flexion (90°)	Dosal†/LS	Yes
Cipolletta et al. [35]	10–22	Linear	7 ± 1 min	Maximal flexion > 60°	Dosal†/LS, TS	Yes
Cipolletta et al. [36]	10–22	Linear	—	Maximal flexion > 60°	Dosal†/LS, TS	Yes
Yildirim et al. [37]	7–12	Linear	—	Maximal flexion	Suprapatellar*/TS	—

Observed joints: *knee joint, †finger joint, ‡shoulder joint, §hip joint, ||metatarsophalangeal joint. —, data not available. *LS*, longitudinal scan; *TS*, transverse scan

In two of 16 studies on finger joints, there was no description of the observation position or scanning method [10, 26]. The other 14 studies assessed the cartilage from the dorsal aspect. One of these studies included additional observations from the palmar aspect [30]. The joint was positioned at maximal flexion in most studies, and the cartilage of the metacarpal head in the MCP joint and proximal phalanx head in the PIP joint were observed. The MTP joint was observed in the longitudinal section of the plantar foot [31]. All studies involving the knee assessed the femoral condylar cartilage using transverse or both transverse and longitudinal probes over the patella in maximum flexion.

Three studies described the examination times for cartilage assessment: < 5 min for four bilateral second and third finger MCP joints [30], < 5 min for 16 bilateral second to fifth finger MCP and PIP joints [29], and 7 min ± 1 min for eight bilateral second to fifth finger MCP joints [35]. Another study reported an examination time of approximately 5 min per joint for the entire MCP joint, including the cartilage [18]. One study also described the assessment times and found that the quantitative method took significantly longer than the semi-quantitative method for the bilateral second to fifth finger MCP joints (6 vs. 8 min) [36].

### Evaluation methods

Table 3 lists the US evaluation methods used in the studies. US evaluation of the cartilage included 15 studies that measured cartilage thickness [3, 12, 13, 18, 19, 21, 23, 28, 29, 32–37], 10 that assessed it binarily [11, 12, 14, 15, 18, 22, 23, 26, 27, 36], and 15 that assessed it semi-quantitatively [10–13, 16, 17, 20, 24, 25, 27, 30, 31, 34–36]. Ten studies assessed two cartilage evaluation methods [11–13, 18, 23, 27, 28, 34–36], and three examined the relationship between them [34–36].

**Table 3:** Evaluation methods

Author	Evaluation methods			Measurement methods		Scoring methods	
	Measurement (quantitative)	Binary	Semi-quantitative	Include the chondrosynovial interface	Sound velocity correction		
Aisen et al. [12]	Yes	—	Yes	—	—	Grades 0–6	Original
Iagnocco et al. [28]	Yes	Yes	—	—	—	Surface irregularity	
Grassi et al. [18]	Yes (MCP 3 in healthy)	Yes	—	—	—	Loss of definition	
Østergaard et al. [19]	Yes	—	—	—	Yes	—	
Lund et al. [20]	—	—	Yes	—	—	Grades 0–3	Original
Batalov et al. [13]	Yes	—	Yes	—	—	Grades 0–6	Original
Möller et al. [29]	Yes	—	—	—	—	—	
Darweesh et al. [21]	Yes	—	—	—	—	—	
Sanja et al. [22]	—	Yes	—	—	—	Cartilage reduction	
Filippucci et al. [30]	—	—	Yes	—	—	Grades 0–4	[38]
Riente et al. [14]	—	Yes	—	—	—	Cartilage changes	
Yücesoy et al. [23]	Yes	Yes	—	—	—	Irregularity, loss of clarity	Original
Di Geso et al. [15]	—	Yes	—	—	—	Presence or absence	
Pereira et al. [24]	—	—	Yes	—	—	Scores 0–4	[38]
Bisi et al. [25]	—	—	Yes	—	—	Grades 0–4	[38]
Mandl et al. [3]	Yes	—	—	Yes	Yes	—	
Onodera et al. [31]	—	—	Yes	—	—	Grades 1–6	[39]
Luz et al. [11]	—	Yes	Yes	—	—	Grades 0–4/presence or absence	[38]
Mesci et al. [32]	Yes	—	—	—	No	—	
Sakthiswary et al. [33]	Yes	—	—	—	No	—	
Sarzi-Puttini et al. [10]	—	—	Yes	—	—	Grades 0–4	[38]
Hurnakova et al. [16]	—	—	Yes	—	—	Grades 0–4	[38]
Yang et al. [26]	—	Yes	—	—	—	Presence or absence	
Mandl et al. [17]	—	—	Yes	—	—	Grades 0–2	[17]
Abda et al. [27]	—	Yes	Yes	—	—	Grades 0–3/presence or absence	
Ogura et al. [34]	Yes	—	Yes	Yes	No	Grades 0–2	[17]
Cipolletta et al. [35]	Yes	—	Yes	Yes	No	Grades 0–4	[38]
Cipolletta et al. [36]	Yes	Yes (quantitative score)	Yes	Yes	No	Grades 0–2	[17]
Yildirim et al. [37]	Yes	—	—	No	No	—	

—, data not available

Nine studies assessed knee thickness [12, 13, 19, 21, 23, 28, 32, 33, 37], and six assessed the fingers (two for the MCP joints [35, 36], three for the MCP and PIP joints [3, 29, 34], and one for MCP-only assessment in healthy participants [18]). Four studies included cartilage measurements from the bone surface to the white band of the chondrosynovial interface for thickness measurements [3, 34–36], one study excluded the white band and measured only the low-echo portion [37], and the other ten studies had no clear description of the thickness. Sound velocity correction was performed in only two cases [3, 19]. Two of the nine studies that measured knee cartilage thickness measured at two locations (medial and lateral) [21, 23], whereas the others are measured at the medial, central, and lateral sites [12, 13, 19, 28, 32, 33, 37].

Three studies used a 3-point scale of 0–2 in the semi-quantitative method [17, 34, 36]. The scale was developed by Taskforce of the Outcome Measures in Rheumatology Ultrasound Working Group (OMERACT US WG) [17]. Seven studies [10, 11, 16, 24, 25, 30, 35] used a 5-point scale of 0–4, which was developed by Disler et al. [38]. One study [31] used a 6-point grading scale of 1–6, based on the grading for OA by Lee et al. [39], and three studies used the original grading.

### Reliability

Table 4 summarizes the inter- and intra-observer reliabilities. Eight studies assessed reliability, all of which involved the finger joint [3, 11, 17, 25, 29, 30, 34, 35]. Four studies focused on the assessment of reliability as the main objective [17, 25, 29, 30]. Reliability can be assessed in two ways: use of recorded static images and actual patient examinations.

**Table 4:** Reliability and validity

Author	Reliability		Validity			
	Interobserver	Intraobserver	Association with other methods	Association with patient data	Association with other participants	Association between evaluation methods
Aisen et al. [12]	—	—	—	—	No	No
Iagnocco et al. [28]	—	—	—	—	Yes	No
Grassi et al. [18]	—	—	—	—	Yes	No
Østergaard et al. [19]	—	—	MRI	—	—	—
Lund et al. [20]	—	—	—	—	Yes	—
Batalov et al. [13]	—	—	—	—	No	No
Möller et al. [29]	Yes	Yes	CR (JSN, JSW)	Yes	Yes	—
Darweesh et al. [21]	—	—	—	Yes	No	—
Sanja et al. [22]	—	—	—	—	Yes	—
Filippucci et al. [30]	Yes	—	CR (JSN)	—	—	—
Riente et al. [14]	—	—	—	—	—	—
Yücesoy et al. [23]	—	—	MRI	—	—	No
Di Geso et al. [15]	—	—	—	Yes	—	—
Pereira et al. [24]	—	—	—	Yes	—	—
Bisi et al. [25]	Yes	—	—	—	—	—
Mandl et al. [3]	Yes	Yes	CR (JSN, JSW)	Yes	—	—
Onodera et al. [31]	—	—	In vivo US, histologic	—	—	—
Luz et al. [11]	Yes	—	—	Yes	—	—
Mesci et al. [32]	—	—	—	Yes	Yes	—
Sakthiswary et al. [33]	—	—	—	Yes	Yes	—
Sarzi-Puttini et al. [10]	—	—	—	—	—	—
Hurnakova et al. [16]	—	—	CR (JSN)	Yes	—	—
Yang et al. [26]	—	—	—	Yes	No	—
Mandl et al. [17]	Yes	Yes	—	—	—	—
Abda et al. [27]	—	—	—	Yes	—	No
Ogura et al. [34]	Yes	Yes	CR (JSN)	Yes	Yes	Yes
Cipolletta et al. [35]	Yes	Yes	—	—	—	Yes
Cipolletta et al. [36]	—	—	CR (JSN)	Yes	Yes	Yes
Yildirim et al. [37]	—	—	—	Yes	Yes	—

Yes, compared in the article; no, not compared in the article; —, data not available. *CR*, conventional radiography; *JSN*, joint space narrowing; *JSW*, joint space width; *MRI*, magnetic resonance imaging; *US*, ultrasonography

In the static images, the interobserver reliability of the three evaluators in the 0–4 semi-quantitative method assessment was prevalence-adjusted bias-adjusted kappa = 0.58, 0.63 (MCP 2 and 3, respectively) [25], while the interobserver reliability of the two evaluators was kappa = 0.82 (MCP 2 and 3) [11]. In the three-grade semi-quantitative evaluation of 0–2 which was proposed by OMERACT US WG with 17 evaluators, interobserver reliability was light’s kappa = 0.64, and intraobserver reliability was weighted kappa = 0.87 [17]. In another study using this method, the interobserver reliability was Krippendorff’s alpha = 0.60 with three evaluators, and the intraobserver reliability was = 0.81 with one evaluator [34].

In a study of patient assessments, the interobserver reliability for two examiners based on the 0–4 semi-quantitative assessments was weighted as kappa = 0.672 and 0.832 for MCP joints 2 and 3 on the palmar and dorsal sides, respectively [30]. In another study, two examiners had interobserver reliability of Cohen’s kappa = 0.66 and intraobserver reliability = 0.73 at MCP joints 2–5 [35]. In an OMERACT US WG three-stage semi-quantitative evaluation by 12 examiners, interobserver reliability for MCP joints 2–5 was light’s kappa = 0.48, intra-observer reliability was weighted kappa = 0.83, while for PIP, the values were 0.17 and 0.66, respectively [17].

On the other hand, two studies showed inter- and intra-observer reliability values of intraclass correlation coefficients (ICC) = 0.80–0.93 and 0.78–0.93, respectively, in the quantitative evaluation of MCP by two examiners [3, 35]. In one of these studies, the smallest detectable difference (SDD) was 0.09 mm (22-MHz probe) for inter- and intra-observer reliability [35]. For a total of 16 MCP and PIP joint scores, inter- and intra-observer reliability were *ICC* = 0.844 and 0.928, respectively, and inter-observer *SDD* = 0.09 mm [29].

### Validity

Table 4 shows the association between the US and other findings. Nine studies compared US with other methods of cartilage assessment, one compared US semi-quantitative methods to histological grade [31], six compared US to CR [3, 16, 29, 30, 34, 35], and two compared US to MRI [19, 23]. One study compared US and anatomical thickness in healthy participants [3]. The ultrasonographic and anatomical thicknesses of five healthy cadaveric MCP joint specimens were compared. Significant differences were not observed in measuring cartilage thickness on cadaveric specimens between the anatomical and US methods (0.67 mm vs. 0.69 mm), and the ICC between the two measurements showed moderate agreement (0.61; 95% *CI*, 0.23–0.83).

Another study assessing semi-quantitative methods and histological grading was performed on the MTP joint; six grades of US findings were compared with four histological grades, and a significant correlation was found (Spearman’s correlation coefficient, rho = 0.67).

In comparison with CR, all six studies examined the fingers. Three of these studied the MCP and PIP joints [3, 29, 34], whereas the remaining three studied the MCP joint alone [16, 30, 35]. There were comparisons between cartilage thickness measured using US and JSN [3, 29, 34, 36], cartilage thickness measurement and joint space width (JSW) [3, 29], and semi-quantitative methods measured using US and JSN [16, 30, 34] in four, two, and three studies, respectively. All studies showed a significant correlation between the US and CR assessments.

Two studies that compared MRI and US evaluated the knees. In one study, both methods yielded high correlations in the measurement of cartilage thickness, with no systematic error in the difference between them [19]. However, in another study, US measurements were significantly thicker than the MRI measurements. However, there was significant agreement between the two morphological assessments [23].

### Correlation with clinical data

Fourteen studies examined the associations between clinical and demographic data [3, 11, 15, 16, 21, 24, 26, 27, 29, 32–34, 36, 37] (Table 5). The main factors examined were age, sex, disease duration, and disease activity. The most significant association was with disease duration, which was found in four of the six studies [16, 29, 34, 36]. Disease activity [26, 37] and age [15, 16] were significantly associated in two out of five studies.

**Table 5:** Correlation with clinical data

Author	Patient characteristics						Global assessment		laboratory data				
	Age	Sex	Disease duration	Height/weight	BMI	TJC/SJC	Disease activity	HAQ	CRP	ESR	RF/anti-CCP	Comp	Others
Aisen et al. [12]	—	—	—	—	—	—	—	—	—	—	—	—	—
Iagnocco et al. [28]	—	—	—	—	—	—	—	—	—	—	—	—	—
Grassi et al. [18]	—	—	—	—	—	—	—	—	—	—	—	—	—
Østergaard et al. [19]	—	—	—	—	—	—	—	—	—	—	—	—	—
Lund et al. [20]	—	—	—	—	—	—	—	—	—	—	—	—	—
Batalov et al. [13]	—	—	—	—	—	—	—	—	—	—	—	—	—
Möller et al. [29]	Yes (healthy)	Yes (healthy)	Yes	Yes (height)/no (weight) (healthy)	No (healthy)	—	—	—	—	—	—	—	—
Darweesh et al. [21]	—	—	—	—	—	—	—	—	—	—	—	Yes (synovial)	—
Sanja et al. [22]	—	—	—	—	—	—	—	—	—	—	—	—	—
Filippucci et al. [30]	—	—	—	—	—	—	—	—	—	—	—	—	—
Riente et al. [14]	—	—	—	—	—	—	—	—	—	—	—	—	—
Yücesoy et al. [23]	—	—	—	—	—	—	—	—	—	—	—	—	—
Di Geso et al. [15]	Yes	—	No	—	Yes	—	No	No	No	No	No	—	Yes (Lequesne index, pain)
Pereira et al. [24]	—	—	—	—	—	—	—	—	—	—	—	—	Yes (pain)
Bisi et al. [25]	—	—	—	—	—	—	—	—	—	—	—	—	—
Mandl et al. [3]	—	—	—	—	—	No	No (SDAI)	—	No	—	—	—	—
Onodera et al. [31]	—	—	—	—	—	—	—	—	—	—	—	—	—
Luz et al. [11]	—	—	—	—	—	—	No (DAS28-ESR)	No	No	No	—	—	—
Mesci et al. [32]	No	—	No	—	—	—	No (DAS28-ESR)	No	No	No	—	—	—
Sakthiswary et al. [33]	—	—	—	—	—	—	—	—	—	—	—	Yes (serum)	—
Sarzi-Puttini et al. [10]	—	—	—	—	—	—	—	—	—	—	—	—	—
Hurnakova et al. [16]	Yes	No	Yes	—	No	—	—	—	—	—	No	—	—
Yang et al. [26]	—	—	—	—	—	—	Yes	Yes	—	—	Yes	—	—
Mandl et al. [17]	—	—	—	—	—	—	—	—	—	—	—	—	—
Abda et al. [27]	—	—	—	—	—	—	—	—	—	—	—	—	Yes (grip strength)
Ogura et al. [34]	No	—	Yes	No	No	—	No (DAS28-CRP)	No	No (CRP)	—	—	—	—
Cipolletta et al. [35]	—	—	—	—	—	—	—	—	—	—	—	—	—
Cipolletta et al. [36]	No	No	Yes	No	No	—	No (CDAI)	—	No (CRP)	—	No	—	—
Yildirim et al. [37]	No	—	No	No	No	Yes (TJC)/no (SJC)	Yes (DAS28-ESR/CRP)	—	—	—	No	—	—

—, data not available. *anti-CCP*, anti-cyclic citrullinated peptide antibody; *BMI*, body mass index; *CDAI*, clinical disease activity index; *COMP*, compared cartilage oligomeric matrix protein; *CRP*, c-reactive protein; *DAS28*, disease activity score; *ESR*, erythrocyte sedimentation rate; *HAQ*, health assessment questionnaire; *RF*, rheumatoid factor; *SDAI*, simplified disease activity index; *SJC*, swollen joint count; *TJC*, tender joints count

Two studies compared cartilage oligomeric matrix protein (COMP) levels in serum or synovial fluid, both in the knee cartilage; one study found a correlation with serum [33], and another study found a correlation with COMP in synovial fluid, but not in the serum [21].

### Temporal changes in the cartilage

Two studies assessed changes over time; one assessed the changes in the cartilage over time in six MCP joints using a semi-quantitative assessment (0–4) in a 52-week prospective study examining the prediction of certolizumab pegol treatment response [10]. The modified total Sharp score over 52 weeks was almost constant, and cartilage assessment showed no significant differences. A study validating a comprehensive ultrasonographic scoring system included the cartilage assessment of four MCP joints over 12 months [11]. In a cohort of untreated patients with RA, with approximately half eventually using biologics, there was an increase in the mean semi-quantitative (0–4) scores, but no significant change. In contrast, the binary evaluation (0 or 1), which divided the semi-quantitative scores into two groups, showed a significant increase in mean scores.

## Discussion

To date, systematic literature reviews of ultrasonographic cartilage assessment in RA have included studies conducted by the OMERACT US WG for the development of a semiquantitative US scoring system [17] and assessment of the evidence for the use of US in structural joint damage in patients with RA [5].

This scoping review provides an overview of ultrasonographic cartilage evaluation in RA, emphasizing ultrasonographic cartilage evaluation, updating the literature, including studies using newly developed semiquantitative evaluation methods, and identifying research gaps.

The extracted articles included those reported between 1984 and 2022, each with a different historical background, including changes in the RA classification criteria and technical differences due to advances in US equipment. They also acknowledged the high heterogeneity, including differences in the characteristics of the participating patient populations and statistical analysis methods. Regarding the knee and finger joints, which were mainly assessed, the cartilage of the bone on the proximal side of the joint was evaluated with the joint in maximum flexion in almost all cases where the limb position of observation was specified. The knee was evaluated from the suprapatellar margin. However, the usefulness of observation from the parapatella was also noted in OA [40], and which site is more suitable for measurement in RA assessment is yet to be determined. There are two main evaluation methods: quantitative evaluation, which measures the thickness of the cartilage, and binary or semiquantitative evaluation, which visually evaluates the white band of the chondrosynovial interface, thickness, and other parameters. Despite advances in ultrasound technology, these two methods have been used for many years, and no new evaluation methods have been presented. However, these methods show heterogeneity.

A review of ultrasonographic cartilage measurements in OA pointed out the following caveats for cartilage thickness measurements: correction owing to the ultrasound propagation velocity of the cartilage, vertical ultrasound beam incidence angle, and inclusion of the white band of the chondrosynovial interface [41].

US equipment measures the distance based on an average propagation velocity of 1540 m/s in the tissue; however, cartilage is known to have a high propagation velocity owing to its stiffness. Thus, it is necessary to correct for sound velocity for more accurate cartilage thickness measurements. However, damaged cartilage is known to have a slower propagation velocity, and whether sound velocity correction is necessary for patients with RA or there is an appropriate correction speed is unknown.

In addition, if the incident angle of the ultrasound is oblique to the cartilage, the refraction of the ultrasound leads to measurement errors, and the white band is unclear. Particularly in the case of the knee, the cartilage is mainly delineated in the transverse image, and cartilage thickness is measured at two or three locations (medial/lateral and midline). However, the ultrasound beam is not incident at a right angle, especially for medial and lateral measurements. Despite these issues, few studies have described the US technique well, and there are insufficient details to compare the studies.

Reliability assessments of measurements included only three studies on MCP joints, each examined by acquiring images from patients by three examiners in a single-center study [3, 29, 35]. These studies showed relatively high inter- and intra-examiner reliabilities and are considered feasible; however, there are no data for the other joints. Furthermore, assessment time is important for quantitative methods when considering feasibility, but only one study has examined the time required for quantitative and semiquantitative assessments [35]. Simplifying the evaluation, including automatic measurements, is an issue for future studies.

The most commonly used semiquantitative method is the 5-point grading of 0–4 by Disler et al. [38]. The reliability used in patients shows moderate-to-high inter- and intra-examiner reliabilities, indicating feasible reliability. The reliability of these studies was evaluated by up to three examiners.

The OMERACT task force examined the reliability of the semiquantitative method of cartilage assessment in OA on a 4-point grading of 0–3 in 10 examiners and found insufficient agreement, particularly poor reliability of the intermediate scores [42].

Therefore, OMERACT has recently advocated a 3-point grading of 0–2 for cartilage assessment in RA [17].

This method is considered well validated in reliability assessments, with 17 participants in assessments from static image readings and 12 from patient examinations, and reliable based on other studies from different regions [34]. However, although this method has achieved feasible reliability for MCP joints, it is not sufficiently reliable for PIP. In addition, most semi-quantitative evaluations have examined finger joints, and many studies have only been binary for the knee. The semi-quantitative evaluation of the joints other than the MCP joints must be explored in future studies.

Validity comparisons with the anatomical measurements of the finger and knee joints’ cartilage have been reported and validated [3, 43]. Semi-quantitative methods have also shown a correlation with pathology in MTP joints, and in vitro studies have shown that damaged cartilage can be detected by US [31].

However, in vitro studies have evaluated artificially damaged cartilage and may differ from cartilage changes in actual patients with RA. In addition, contrasting US findings with anatomical thickness and tissue findings is limited to cadavers and surgical tissue collection, which makes it difficult to contrast the state of the cartilage early in the disease, which biases the research.

In this respect, there have recently been several attempts to assess cartilage composition in MRI using various imaging methods and capture qualitative changes in the cartilage before morphological changes [44, 45]. However, only two comparisons between US and MRI were performed, which only measured cartilage thickness in the knee cartilage. A comparison with joints other than the knee and cartilage composition assessments is a subject for future investigation.

By contrast, cartilage damage in imaging examinations is generally based on JSN by CR, albeit an indirect assessment, and JSN scoring in patients with RA is still widely used [46, 47]. Each ultrasound evaluation in the articles extracted showed a significant correlation with the JSN score based on CR. These results demonstrate the validity of US cartilage assessment and show that JSN assessment using CR reflects cartilage damage. Therefore, the added value of cartilage assessment by US over that by CR needs to be investigated.

JSW remains generally unaccepted in CR, although it has been suggested to be a more reliable assessment than the semi-quantitative method of JSN scoring [48]. This is because both JSN and JSW assess the joint gap width, and semi-quantitative assessment is simpler than quantitative assessment and currently sufficiently sensitive and reliable. However, the semi-quantitative method in US may have a different meaning than the quantitative method because of factors other than cartilage thickness, such as opacification of the outer edge of the cartilage and localized changes.

In fact, the report suggests that the OMERACT grading of RA in older age groups shows more changes than the quantitative assessment, and the semi-quantitative assessment may be more likely to show changes in age and OA. However, the report also stated that more joints in the younger age group had findings on the quantitative assessment than in the older age group, although the semi-quantitative assessment showed no problems. Therefore, the quantitative assessment might be more likely to detect cartilage changes in RA on US examination [36]. However, whether the pathology and assessment methods make a difference requires further investigation.

Furthermore, advances in treatment have reduced the number of structural damage changes, and clinical trials are increasingly incorporating MRI, which can rapidly detect changes in conjunction with ethical factors [49, 50]. Similarly, US, which can directly assess cartilage, has the potential to detect earlier and more subtle changes than CR assessment; however, these data are lacking. Compared with healthy participants and patients with other diseases in terms of discriminant validity, significant cartilage thickness thinning and increased semi-quantitative scores were found in patients with RA compared with healthy participants. There are also reports of a significant correlation between cartilage damage and disease duration, suggesting that cartilage damage can be assessed using US as the disease progresses. However, a comparison of cartilage thickness in patients with early onset RA and healthy participants reported no significant differences in MCP joints but significantly thinner cartilage in the knee joints. Each study showed differences, and further research is warranted. Moreover, there have been no comparisons between cartilage damage with OA or other inflammatory joint diseases.

Finally, their reliability and validity must be verified through a longitudinal assessment. However, data on the longitudinal evaluation of the cartilage using US are minimal, which is the most important issue when considering the usefulness of US.

As described above, US recognizes many issues in cartilage evaluation. Although it is currently considered a valuable method for directly assessing cartilage, the lack of data regarding the significance of cartilage assessment by US is particularly a major hindrance for its effective use in daily clinical practice. However, direct evaluation of cartilage damage is important under the current treatment strategy, and US, which can be easily performed, is expected to become an important evaluation method.

Nevertheless, our study has some limitations. This study only included searches for peer-reviewed articles published in English, which may not have included some studies or the most recent results. Additionally, we did not check the quality of the literature to map a wide range. Furthermore, the results for each item were not integrated, indicating that the results should be evaluated with caution.

## Conclusions

Many aspects of the ultrasonographic cartilage assessments in patients with RA were heterogenous. Most current studies are limited to the fingers and knees, and although quantitative and semi-quantitative assessments are mainly performed, the methods and assessments used were heterogeneous. The reliability and validity of each method suggest the usefulness of US for cartilage assessment; however, it is limited to localized areas and requires further evaluation. Moreover, data on which method is more useful and needs to be included in longitudinal assessments are lacking.

Furthermore, the validity of the commonly used JSN with CR has been recognized; however, additional value-added data by US are lacking. The usefulness of this method for detecting early and subtle changes that are difficult to detect with JSN and assessing cartilage quality requires further examination.

## Supplementary Information

**Additional file 1.** Supplementary Data. Search formulae.

## Data Availability

The data are available in the article, and further inquiries can be directed to the corresponding authors.

## Abbreviations

COMP: Cartilage oligomeric matrix protein
CR: Conventional radiography
ICC: Intraclass correlation coefficients
JSN: Joint space narrowing
JSW: Joint space width
MCP: Metacarpophalangeal
MRI: Magnetic resonance imaging
MTP: Metatarsophalangeal
OA: Osteoarthritis
OMERACT US WG: Outcome Measures in Rheumatology Ultrasound Working Group
PIP: Proximal interphalangeal
RA: Rheumatoid arthritis
SDD: Smallest detectable difference
US: Ultrasonography
