# Supplementary material for: Cartilage evaluation by ultrasonography in patients with rheumatoid arthritis: a scoping review
Source: Inflamm Regen. 2023 Jul 4;43:34. doi: 10.1186/s41232-023-00286-2 (PMC10318783; doi:10.1186/s41232-023-00286-2)
Supplement: Supplementary file 1 — Additional file 1. Supplementary Data. Search formulae. [file 41232_2023_286_MOESM1_ESM.pdf]

Supplementary Data. Search formulae

Database: PUBMED

Date: 2022/7/23

#	Search terms	Number of hits
1	"arthritis, rheumatoid"[MH]	122,042
2	"rheumatoid arthritis"[tiab]	116,617
3	"arthritis"[tiab] AND "rheumatoid"[tiab]	123,022
4	"RA"[tiab]	86,509
5	#1 OR #2 OR #3 OR #4	196,932
6	"cartilage"[MH]	86,319
7	"cartilage*"[tiab]	96,591
8	#6 OR #7	139,848
9	"ultrasonography"[MH]	476,628
10	"ultrasonics"[MH]	25,448
11	"ultrasound*"[tiab]	293,842
12	"ultrasonograph*"[tiab]	122,677
13	"ultrasonic*"[tiab]	64,006

14	"sonograph*"[tiab]	58,360
15	"US"[tiab]	503,411
16	#9 OR #10 OR #11 OR #12 OR #13 OR #14 OR #15	1,176,091
17	#5 AND #8 AND #16	207

Database: EMBASE

Date: 2022/7/29

#	Search terms	Number of hits
1	rheumatoid arthritis'/exp	240,565
2	rheumatoid arthritis':ti,ab,kw	180,033
3	#1 OR #2	264,129
4	cartilage'/exp	158,500
5	cartilage:ti,ab,kw	121,903
6	#4 OR #5	198,612
7	echography'/exp	964,906
8	ultrasound'/exp	212,789
9	ultrasonograph*':ti,ab,kw	177,967
10	ultrasound*':ti,ab,kw	463,607

11	ultrasonic*':ti,ab,kw	74,816
12	sonograph*':ti,ab,kw	82,914
13	echogra*':ti,ab,kw	14,544
14	#7 OR #8 OR #9 OR #10 OR #11 OR #12 OR #13	1,315,076
15	#3 AND #6 AND #14	468

Database: COCHRANE

Date: 2022/7/23

#	Search terms	Number of hits
1	("rheumatoid arthritis"):ti,ab,kw	16,818
2	(cartilag*):ti,ab,kw	3,631
3	[mh Ultrasonography]	14,868
4	(ultrasound*):ti,ab,kw	35,379
5	(ultrasonograph*):ti,ab,kw	18,231
6	(echogra*):ti,ab,kw	6,813
7	#3 OR #4 OR #5 OR #6	51,869
8	#1 AND #2 AND #7	12
